# Short term outcome of congenital hyperinsulinism: case series

**DOI:** 10.1186/1687-9856-2013-S1-P176

**Published:** 2013-10-03

**Authors:** Song Hai Lim, Janet YH Hong, MZ Fuziah, S Flanagan, S Ellard, K Hussain

**Affiliations:** 1 Endocrine Unit, Paediatric Department, Putrajaya Hospital, Malaysia; 2 Molecular Genetics Laboratory, Royal Devon and Exeter NHS Healthcare Trust, Exeter, UK

## Introduction

Congenital hyperinsulinism (CH) is a major cause of recurrent hypoglycaemia in neonates and infants, results in varying degrees of neurological impairment. Advances in molecular study and imaging technique have been used to guide treatment option for CH. However, there were centres treating CH with long-term medications and reported good neurodevelopmental outcome.

## Objective

To describe short-term outcome of infants with CH in Putrajaya Hospital, Malaysia.

## Method

Medical data of all patients diagnosed with CH between 15 October 2007 and 31 December 2011 was retrieved from electronic medical record. Their clinical features and treatment outcome were reviewed. Genetic study was performed in Exeter, United Kingdom.

## Results

Five infants were reported. Hypoglycaemic seizure was the commonest presenting feature. All had detectable insulin level (>5 mU/l) during hypoglycaemic episodes and high glucose requirement (>10 mg/kg/min). Three patients were found to have genetic mutation associated with CH. Patient 1 had hyperammonaemia hyperinsulinism syndrome and corresponded missense mutation of *GLUD1* gene. For past 2.5 years, there was good response to diazoxide treatment. Patient 2 had diffuse disease with homozygous mutation at the *ABCC8* gene. She presented at birth with severe disease and required combination of medications including octreotide. It was complicated by epilepsy, and developmental milestones were mildly delayed. Patient 3 inherited a heterozygous mutation in the *KCNJ11* gene from the father (presumed focal disease). He responded only to octreotide and showed normal development at nine months old. Another two patients had no common mutation detected. Patient 4 required combination of therapy initially, but subsequently treated with diazoxide alone and weaned off nasogastric feeding at five years old. Patient 5 had features suggestive of Beckwith-Wiedemann syndrome and did not respond to all medical therapy. Pancreatectomy was performed at 3 months old and he died from complications of surgery.

## Conclusion

Molecular genetic study is useful in the management of neonates and infants with CH. Patients with focal and diffuse disease were shown to respond to medical therapy.

